# Brain structural and functional differences between pure menstrual migraine and menstrually-related migraine

**DOI:** 10.1038/s41598-020-73399-0

**Published:** 2020-10-05

**Authors:** Tao Xu, Yutong Zhang, Chen Wang, Huaqiang Liao, Siyuan Zhou, Dehua Li, Siying Huang, Yu Shi, Ziwen Wang, Jiao Chen, Fan-Rong Liang, Ling Zhao

**Affiliations:** 1 College of Acupuncture and Tuina, Chengdu University of Traditional Chinese Medicine, 37 Shierqiao Road, Chengdu, Sichuan China; 2 School of Life Science and Technology, Xidian University, Xi’an, China; 3 MRI Center, The Hospital of Chengdu University of Traditional Chinese Medicine, Chengdu, China

**Keywords:** Migraine, Migraine

## Abstract

The pathophysiological differences between menstrually-related migraine (MRM) and pure menstrual migraine (PMM) are largely unclear. The aim of this study was to investigate the potential differences in brain structure and function between PMM and MRM. Forty-eight menstrual migraine patients (32 MRM; 16 PMM) were recruited for this study. Voxel-based morphometry (VBM) was applied on structural magnetic resonance imaging (sMRI), and the amplitude of low-frequency fluctuations (ALFF) and regional homogeneity (ReHo) in resting state functional MRI (rsfMRI) were calculated. No significant between-group difference was observed in the grey matter volume (GMV). MRM patients exhibited lower ALFF values at the dorsolateral prefrontal cortex (DLPFC) and medial prefrontal cortex (mPFC) than PMM patients. Moreover, the MRM group showed significantly higher ReHo values in the DLPFC. Higher values in the mPFC were related to higher expression of calcitonin gene-associated peptide (CGRP) in the PMM group (r = 0.5, *P* = 0.048). Combined ALFF and ReHo analyses revealed significantly different spontaneous neural activity in the DLPFC and mPFC, between MRM and PMM patients, and ALFF values in the mPFC were positively correlated with CGRP expression, in the PMM group. This study enhances our understanding of the relationship between neural abnormalities and CGRP expression in individuals with PMM.

## Introduction

According to the International Classification of Headache Disorders III beta (ICHD-III beta), menstrual migraine (MM) attacks are associated with the menses and are divided into two subcategories: pure menstrual migraine (PMM) and menstrually-related migraine (MRM)^1,2^ Unlike PMM, MRM involves migraine attacks that occur outside of the menstrual cycle. Differences between the incidence of PMM and MRM have been observed. An epidemiological survey showed that 21% of women with migraine experience MM, and approximately 66% experience MRM^3^. Triptans are the best, proven, first-line treatment for MRM and PMM, but it has been reported that MRM is less responsive to this treatment^4^. In light of this difference in treatment response, it is highly likely that pathophysiological differences between MRM and PMM exist.

Although the precise aetiology remains incompletely understood, an inflammatory response is generally accepted as a possible mechanism for the pathogenesis of MM^5^. The neuropeptide calcitonin gene-related peptide (CGRP), a highly potent vasodilator, is a marker of trigeminal nerve inflammation, and plays an important role in various components of migraine attacks^5^. Serum concentrations of CGRP, which has been regarded as an important medium in the pathophysiology of PMM, are elevated during migraine attacks^5^. However, few studies have characterised the relationship between MRM and CGRP. Additionally, one study provided evidence for disturbed systemic and trigeminovascular cyclicity in MRM patients, and not in PMM patients, which may increase their susceptibility to experiencing migraines during menstruation^6^. Comparison of the pathophysiological mechanisms of MRM and PMM may provide valuable guidance for the development of viable clinical treatment options for MM.

Neuroimaging studies have confirmed that migraine is not only a neurovascular disorder, but also a multifaceted, central nervous system disorder^7^. Alterations in the brain's structure and function due to migraine, have been seen in various regions including the prefrontal cortex, insula, temporal gyrus, posterior cingulate cortex, anterior cingulate cortex, and thalamus^7^. Consistent with these findings, our previous studies have reported that that high-frequency migraine attacks can influence the structural and functional communication patterns of the frontal cortex, and these changes may explain the functional impairments of migraine patients^8^. We conclude that migraine is a progressive disease and that continual experiences may have an additive effect on the structure and function of the brain.

Due to the differences in attack frequency, there is a high possibility that PMM and MRM migraines may have different effects on brain structure and function. Thus, we hypothesised that, in terms of brain structure and function, different central mechanisms exist between PMM and MRM. Resting-state functional MRI (rsfMRI) and structural magnetic resonance imaging (sMRI) were used to investigate any functional and structural changes in the brain between the different patient groups. To test this hypothesis, voxel-based morphometry (VBM) was applied on sMRI^9^, and the amplitude of low-frequency fluctuations (ALFF) and regional homogeneity (ReHo) in rsfMRI were calculated^10,11^.

## Methods

### Study design

This study was approved by the Sichuan Regional Ethics Review Committee on TCM (ethical approval number: 2015KL-004) and registered in the Chinese Clinical Trial Registry (registration number: ChiCTR-IOR-15006648). After fully explaining the experimental procedures, all participants were required to sign written informed consent documents. Participants were enrolled from two hospitals in Chengdu, Sichuan, China: The Hospital of Chengdu University of Traditional Chinese Medicine (TCM) and Chengdu Integrated TCM &Western Medicine Hospital. Recruitment took place from June 2015 through August 2018. All experiments were performed in accordance with the relevant guidelines and regulations.

The diagnosis of PMM and MRM was established according to the ICHD-III beta (https://www.ichd-3.org/) guidelines. The inclusion criteria were as follows: (1) Female, 18–50 years old, right-handed; (2) migraine attacks outside of the menstrual cycle cannot exceed 6 times per month; (3) a history of menstrual migraine without aura for 6 months or more; and (4) a stable 28 (± 7)-day menstrual cycle. Patients with any of the following conditions were excluded: (1) existence of neurological diseases; (2) hypertension, diabetes mellitus, hypercholesterolemia, vascular/heart disease, and any major systemic conditions; (3) pregnancy or lactation; (4) history of alcohol or drug abuse; (5) any neuroimaging research study participation during the last 6 months; and (6) inability to understand the doctor’s instructions.

Thirty-five right-handed MRM patients (33.41 ± 6.23 years; mean age ± SD) and seventeen PMM patients (31.75 ± 7.85 years; mean age ± SD) were recruited for this study. Four patients were excluded from further analysis due to excessive head movement (> 2 mm) during the functional MRI (fMRI) scan. Thus, a total of 48 subjects (32 MRM patients and 16 PMM patients) were included in the final data analyses (Table 1).

**Table 1:** Demographic characteristics of subjects.

Characteristics	MRM (n = 32)	PMM (n = 16)	*p* value
Age (years)	33.41 ± 6.23	31.75 ± 7.85	0.79
Height (cm)	160.38 ± 7.46	161.63 ± 5.32	0.79
Weight (kg)	53.50 ± 6.24	52.3 ± 5.56	0.79
Education (year)	12.03 ± 2.71	12.18 ± 2.22	0.84
Duration (month)	7.83 ± 6.88	6.94 ± 5.82	0.82
Migraine frequency	3.94 ± 2.03	1.81 ± 0.83	0
Migraine intensity	1.89 ± 0.57	2.06 ± 0.57	0.79
CGRP	175.27 ± 32.28	172.62 ± 39.03	0.80
HIT-6 score	65.97 ± 5.48	59.88 ± 9.61	0.13
MPQ score	79.97 ± 19.95	65.44 ± 26.60	0.13

Values are mean ± SD. Data show the headache activity and characteristics of MRM and PMM, respectively. MRM and PMM had similar headache activity at baseline, including the duration, frequency, and intensity. Patients also showed similar HIT-6 and MPQ score characteristics at baseline.

The comparisons of basic information were performed between MRM and PMM group using a two-sample *t-*test.

*MRM* menstrually-related migraine, *PMM* pure menstrual migraine, *CGRP* calcitonin gene-associated peptide, *HIT* the headache impact test, *MPQ* the McGill Pain Questionnaire.

Prophylactic meditations for migraine were stopped 4 weeks before the experiment; however, the participants were allowed to take emergency medicine if their pain was too intense and hard to bear. Detailed information regarding the participants’ drug intake was recorded. All participants had been free from a typical migraine attack for at least 1 week prior to their MRI examination for this study. After scanning, all participants reported that they had not experienced any headaches or migraines and remained awake during the procedure.

### Clinical data collection

Patient information that was collected included age, height, weight, and education level. Participants were instructed to maintain a migraine diary in which they recorded migraine duration, intensity (evaluated on a scale from 0 to 3: 0, no migraine; 1, mild pain; 2, moderate pain; 3, severe pain.), and frequency (number of times in one month). Participants completed two surveys, the Headache Impact Test-6 (HIT-6) questionnaire, which was developed to assess the severity and impact of migraine on daily life, and the McGill Pain Questionnaire (MPQ), which is used to measure the sensory, affective, and evaluative aspects of pain in adults with chronic pain^12,13^. The Chinese version of the MPQ questionnaire has good reliability and validity in China, and the HIT-6 questionnaire was recommended by the Chinese migraine guidelines^14–20^.

### Blood sample collection

Blood samples were collected on the second or third day of the menstrual period window. Only one sample was taken from each patient. Blood samples were collected in the laboratory between 8 and 10 a.m., after overnight fasting. Sterile, sodium heparin-containing tubes were used for sample collection. After clotting at room temperature for 30 min, samples were centrifuged for 15 min at 2000×*g*. Aliquots were immediately stored at − 80 °C. CGRP expression was determined by competition binding assays using a human CGRP-radioimmunoassay (RIA) kit (Phoenix Peptide Pharmaceutical, Belmont, CA;Ref. RK-015-02). Samples were coded and CGRP determinations were assayed blindly with respect to patient identifiers^21^.

### MRI data collection

MRI was performed during the periovulatory phase (days 12–16 of the menstrual cycle). All participants had been free from a typical migraine attack for at least 1 week prior to the MRI examination. MRI data were acquired with a GE Discovery MR750 3.0 T System (General Electric, Milwaukee, WI) with an eight-channel, phased-array head coil. The functional images were obtained with a single-shot gradient-echo echo-planar imaging (GRE-EPI) sequence with the following parameters: repetition time = 2,000 ms; echo time = 25 ms; flip angle = 90°; field of view = 240 × 240 mm; data matrix = 64 × 64; and slice thickness = 3 mm. Structural MR imaging data were acquired using a 3D T1-weighted spoiled gradient-echo sequence with the following parameters: repetition time = 3.7 ms; echo time = 9.2 ms; flip angle = 12°; and voxel-size = 1 × 1 × 1mm^3^. For each subject, a total of 205 volumes were acquired, resulting in a total scan time of 410 s. All participants were instructed to keep their eyes closed and to stay awake during the entire scan session.

### VBM analysis

VBM is an advanced topological structural analysis method that is widely used to assess regional alterations in grey matter (GM)^22^. VBM has been useful for characterising subtle changes in brain structure in a variety of diseases associated with neurological and psychiatric dysfunction^23^. In this study, VBM analysis was performed using Statistical Parametric Mapping 12 (SPM12, https://www.fil.ion.ucl.ac.uk/spm/) and Data Processing & Analysis of Brain Imaging toolbox version 2.3 (DPABI V2.3; https://rfmri.org/dpabi) software. Steps for image processing included: (1) manual reorientation; (2) segmentation into GM, white matter and cerebrospinal fluid; (3) mean template creation, spatial normalisation into the Montreal Neurological Institute template space and modulation to adjust for changes in volume during spatial normalisation; and (4) the GM segments were smoothed with an isotropic Gaussian kernel of 8 mm full-width at half maximum. Smoothed GM segments were entered into a voxel-wise multiple regression analysis (based on the general linear model) to investigate the variability in regional GMV^9,24^.

### ALFF and ReHo analyses

ALFF and ReHo methods are two important methods for depicting the various characteristics of global rsfMRI signals. ALFF measures the intensity of neural activity at the single-voxel level, whereas ReHo measures the neural synchronisation of a given voxel with its neighbouring voxels^25^. fMRI image processing was carried out using SPM12 and DPABI V2.3 software^24^. The first 10 volumes of individual fMRI data were discarded. The remaining volumes were realigned to the first one to correct for head motion. The individual fMRI images were then spatially normalised to the standard template and re-sampled to a 3 × 3 × 3 mm voxel size. The linear trends were regressed and a band-pass filter was applied at 0.01 ~ 0.08 Hz. The ALFF value for each voxel was calculated by averaging the square root of the power spectrum from 0.01 to 0.08 Hz^10^. The ReHo value for each voxel was obtained by calculating Kendall’s coefficient of concordance (KCC) within a cubic cluster size of 27 voxels^11^.

### Statistical analysis

Demographic and clinical variables were compared between the two groups using SPSS Statistics, version 25.0 (IBM Corp., Armonk, N.Y., USA). An independent two-sample *t*-test was used for comparing continuous variables. *P*-values less than 0.05 false discovery rate (FDR) were considered to be statistically significant.

Hypotheses were tested by comparing the ALFF and ReHo values for the two groups using a linear regression model, controlling for age, level of education, headache duration, headache frequency, headache intensity, and attack duration. This analysis is equivalent to a between-group analysis of covariance with baseline values as a covariate. The statistical threshold was set at *P* < 0.05 (FDR corrected).

To identify the relationship between regional ALFF and ReHo abnormalities, a bivariate correlation was performed between these two algorithms. Briefly, the mean ALFF and ReHo values of brain regions with significant differences were individually extracted and correlated with one another.

To investigate the relationship among mean ALFF/ReHo values of the voxels and CGRP expression/HIT-6 score/MPQ score, Pearson correlation analyses were performed in a voxel-wise manner using DPABI V2.3 software. Analyses were adjusted for the same covariates as those controlled for in the between-group tests. The statistical threshold was set at *P* < 0.05 (FDR corrected) to explore the most significant correlations among MR voxels.

## Results

### Subject demographics

PMM and MRM patients did not differ in terms of age, height, weight, or level of education. No significant differences between the two groups were observed in the migraine duration or intensity, HIT-6 score, MPQ score, or CGRP expression level. Patients with MRM had significantly higher migraine frequency than those with PMM (*P* < 0.001;Table 1).

### VBM results

No significant differences were observed in grey matter volume (GMV) between patients with MRM or PMM.

### ALFF and ReHo results

**Figure 1:**
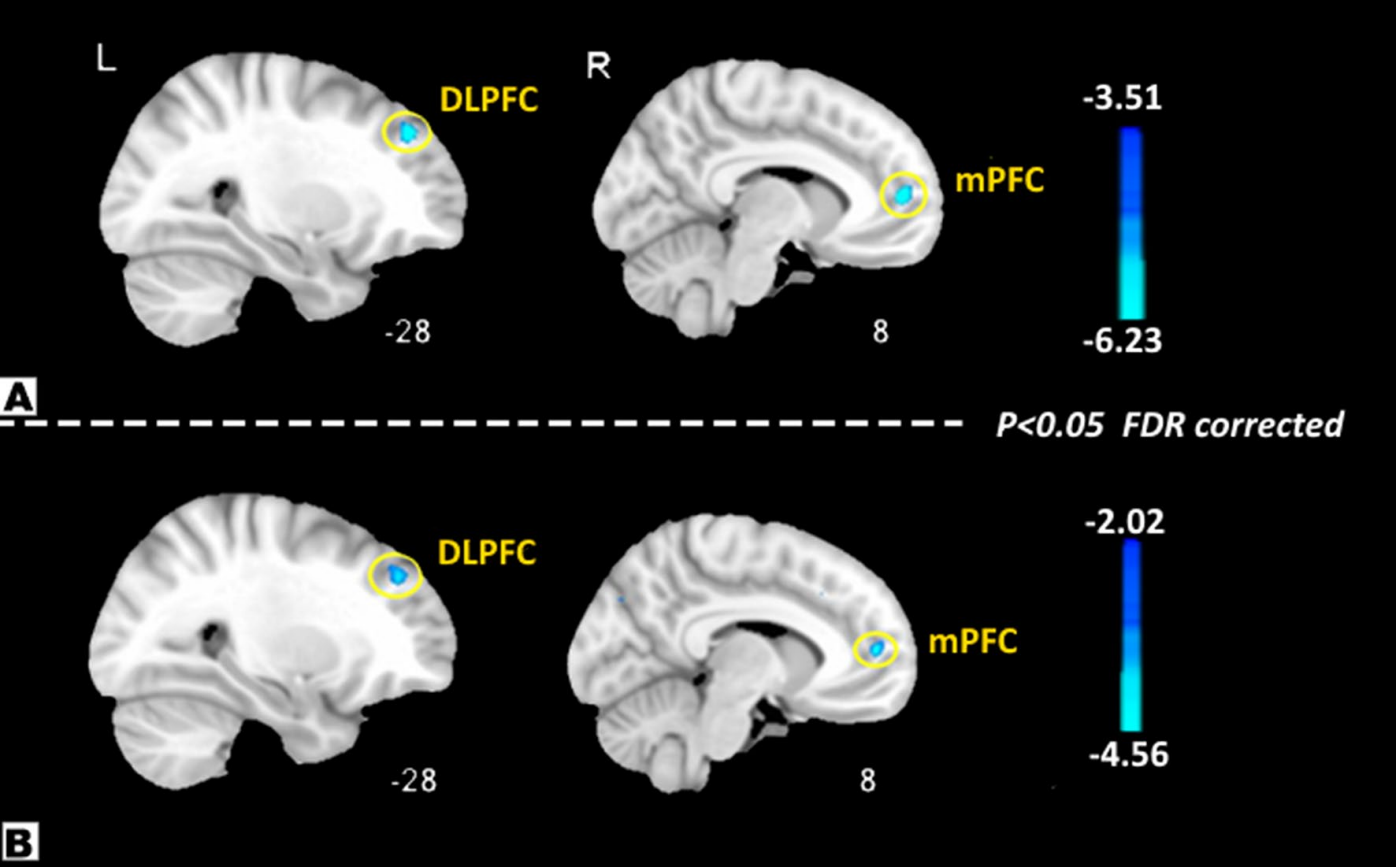
ALFF results of MRM and PMM patients (p < 0.05, FDR corrected). The ALFF differences between the MRM and PMM patients showed significantly lower values in the DLPFC and mPFC (**A**). After controlling for age, level of education, migraine duration, frequency, intensity, and attack duration, compared with patients in the PMM group, MRM patients still showed significantly lower ALFF values in the DLPFC and mPFC (**B**).

**Figure 2:**
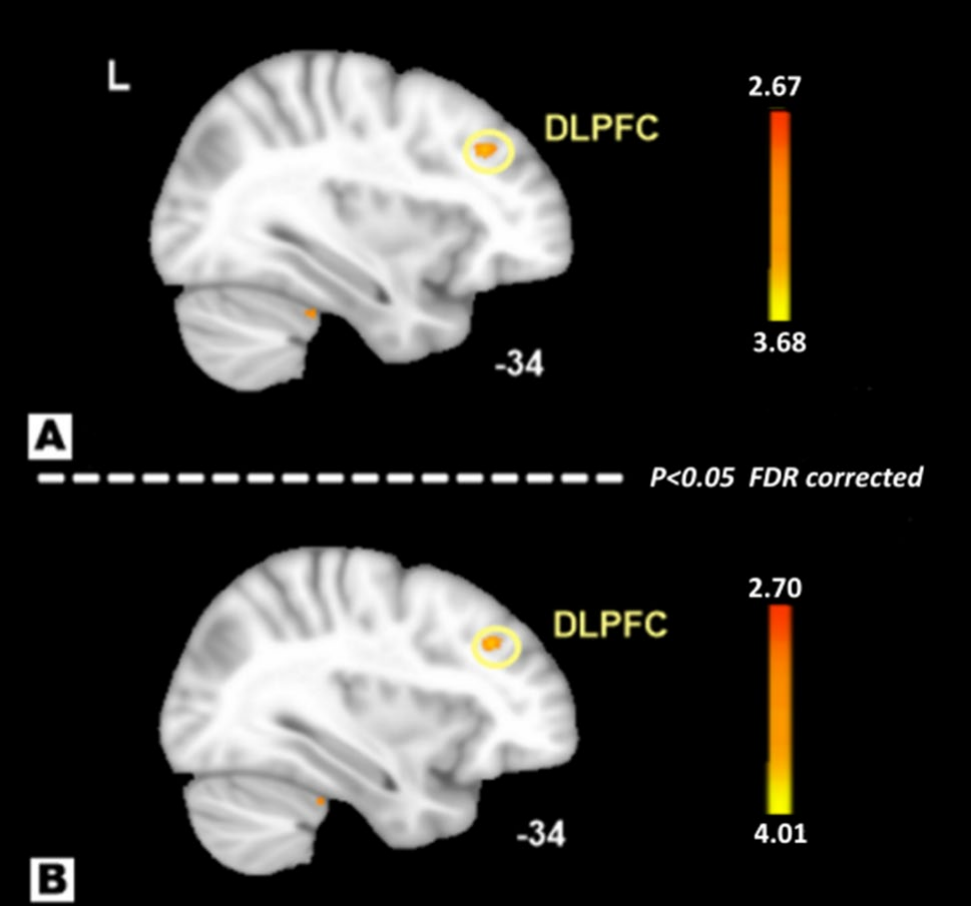
ReHo results of MRM and PMM patients (p < 0.05, FDR corrected). The ReHo differences between MRM and PMM patients showed significantly higher values in the DLPFC (**A**). After controlling for age, level of education, migraine duration, frequency, intensity, and attack duration, compared with patients in the PMM group, MRM patients still showed significantly higher ReHo values in the DLPFC (**B**).

The ALFF values in the dorsolateral prefrontal cortex (DLPFC) and medial prefrontal cortex (mPFC) were lower among patients with MRM than those with PMM (Fig. 1, Table 2). Additionally, patients with MRM had higher ReHo values in the DLPFC compared with PMM patients (Fig. 2, Table 3).

**Table 2:** ALFF difference between MRM and PMM.

Brain region	Side	Cluster size	BA	Voxels with maximum effect			
				Talairach			Peak intensity value
				X	Y	Z	
DLPFC	L	48	9	− 6	47	3	− 6.23
mPFC	R	30	10	30	39	31	− 5.04
**Regression analysis**							
DLPFC	L	16	9	− 42	24	18	− 4.56
mPFC	R	25	10	24	48	22	− 3.97

*DLPFC* the dorsolateral prefrontal cortex, *mPFC* the medial prefrontal cortex, *MRM* menstrually-related migraine, *PMM* pure menstrual migraine, *ALFF* amplitude of low frequency fluctuations.

**Table 3:** ReHo difference between MRM and PMM.

Brain region	Side	Cluster size	BA	Voxels with maximum effect			
				Talairach			Peak intensity value
				X	Y	Z	
DLPFC	L	18	9	− 6	47	3	3.68
**Regression analysis**							
DLPFC	L	21	9	− 39	48	23	4.01

*DLPFC* the dorsolateral prefrontal cortex, *MRM* menstrually-related migraine, *PMM* pure menstrual migraine, *ReHo* regional homogeneity.

### Correlation analysis results

**Figure 3:**
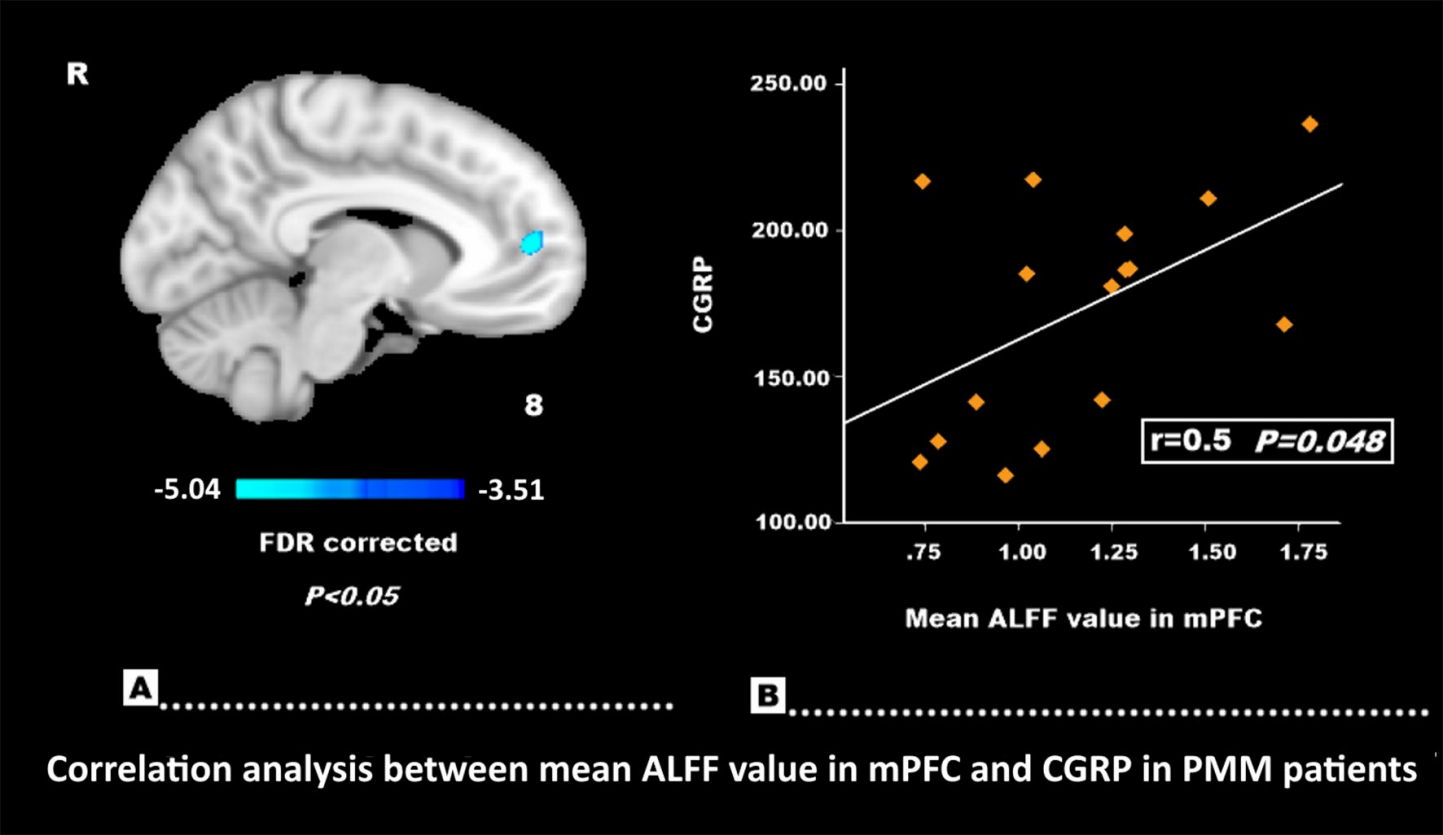
Correlation analysis for CGRP. The mPFC masks extracted values from the ALFF results of the MRM and PMM patients (**A**). ALFF values in the mPFC were positively correlated with CGRP expression in the PMM group (r = 0.5, P = 0.048) (**B**).

ALFF values in the mPFC were positively correlated with CGRP expression in the PMM group (r = 0.5, *P* = 0.048) (Fig. 3). No significant correlations were observed between ALFF and ReHo values (in areas with significant group differences) and HIT-6 or MPQ scores.

## Discussion

This is the first study to explore the differences in brain structure and function between patients with PMM or MRM. No significant difference was noted in GMV between the patients with MRM and PMM. We found that patients with MRM showed lower ALFF values in the DLPFC and mPFC than patients in the PMM group. Moreover, the MRM group displayed significantly higher ReHo values in the DLPFC. In the correlation analysis, higher values in the mPFC were related to higher CGRP expression in the PMM group.

A previous study found significantly decreased GMV among migraine patients without aura, in the following five brain regions: the right occipital lobe, dorsal ACC, left mPFC, brainstem, and cerebellum^7^. A systematic review and meta-analysis of GM alterations in migraine suggested that individuals with migraines had concordant decreases in GMV in the bilateral inferior frontal gyri, right precentral gyrus, left middle frontal gyrus, and left cingulate gyrus. GMV of the bilateral inferior frontal gyri, right precentral gyrus, left middle frontal gyrus, and left cingulate gyrus of migraineurs showed a consistent decrease^26^. Given that migraine can cause substantial changes in brain structure, we compared GMV, but did not find any differences, between patients with MRM or PMM**.** Migraine sufferers showed an abnormal distribution pattern of brain morphology, characterised by areas with decreased or increased GMV^27^. We compared two subgroups of MM patients, PMM and MRM; the, only significant difference observed between the two was in the frequency of migraine occurrence, all other clinical symptoms were comparable between the groups.

ALFF and ReHo have been widely used to explore the neuropathology of various neurological and psychological diseases. These two methods are based on different neurophysiological mechanisms; ALFF analysis shows the intensity of regional spontaneous neural activity and ReHo analysis is used to demonstrate neural concordance^25,28,29^. We proposed that brain activity in these specific regions, may function through intricacies not easily identified or characterised by a single method.

The DLPFC and mPFC pertain to the frontal cortex, which is one of the most prominent areas associated with brain activity, in patients with migraine. The DLPFC has been functionally associated with various roles including the inhibitory control of pain pathways, working memory, cognitive flexibility, planning, inhibition, and abstract reasoning^28–31^. Beyond that, DLPFC has been shown to mediate the part of pain processing associated with localisation and encoding of the attended stimulus^32^.

Given the above findings, we speculate the lower level of regional functional neural activity in the DLPFC and mPFC of MRM patients may influence the inhibitory control of pain perception and mediation of additional attacks of migraine outside of the menstrual cycle.

The DLPFC in patients with MRM, simultaneously exhibited decreased ALFF and increased ReHo compared with PMM patients. Previous work suggests that there is a functional correlation between ReHo and ALFF. A blood-oxygen-level dependent time-series study revealed a strong positive correlation between ReHo and ALFF (r = 0.43–0.64), which reflected the temporal synchrony and amplitude fluctuation of spontaneous neuronal activity of a given voxel, respectively^33^. In a study of stroke patients, significant negative correlations were observed between these two measures^34^. Therefore, we speculate that a higher ReHo in the MRM group may be due to additional migraine attacks outside of the menstrual cycle, induced by alterations in spontaneous brain activity (i.e., decreased ALFF), resulting in enhanced synchronisation.

In our study, ALFF values in the mPFC were positively correlated with CGRP expression in PMM patients. Decades of neuroimaging research indicates that the mPFC mediates a wide variety of pain processing phenomena^35,36^. Serum concentrations of the neuropeptide, CGRP, are elevated during migraine attacks and it has long been postulated to play an integral role in the pathophysiology of migraine^37^. The correlation between variations in ALFF activity within the mPFC and changes in CGRP expression may be responsible for the migraine attacks and pain processing differences seen among PMM patients. However, since no significant correlations have been observed between ALFF and ReHo values (in areas with significant group differences) and HIT-6 or MPQ scores, the potential compensatory neural mechanism still requires further investigation.

When considering treatment options for this patient population, it is important to consider that different central mechanisms exist between PMM and MRM and to distinguish PMM from MRM, as the latter includes additional migraine bouts during non-menstrual times of the month, and this may influence choice of treatment.

### Limitations

This study has several limitations. First, this study involved a relatively small sample size, which may have reduced our ability to detect subtler changes between the groups. Second, due to a lack of hormonal testing, participants’ hormone profiles could not be compared. Given the important role of hormones in menstrual migraine, hormone testing should be introduced in future research to explore its relationship with neuroimaging^38^. Third, the absence of a healthy control group prevents us from providing a more in-depth discussion of the differences observed. Fourth, because of the procedural requirements for each measure, the blood samples and MRI scans were not acquired at the same time point, and this can limit some of our assessments on the association between these two variables. Our results should thus be interpreted with caution, and further longitudinal fMRI studies are required to establish and confirm the current findings.

## Conclusions

In summary, our combined ALFF and ReHo analyses revealed a significant difference in spontaneous neural activity in the DLPFC and mPFC between MRM and PMM, Additionally, patients with PMM showed a positive correlation between ALFF values in the mPFC and CGRP expression. This study provided a novel approach for investigating the differences in central mechanisms between patients with MRM or PMM. Furthermore, this work elucidates the relationship between neural abnormalities and CGRP expression in individuals with PMM.

## Data Availability

The processed data required to reproduce these findings cannot be shared at this time as the data also forms part of an ongoing study.

## References

[1] Pavlovic JM (2015). Burden of migraine related to menses: results from the AMPP study. J. Headache Pain.

[2] Headache Classification Committee of the International Headache, S. The International Classification of Headache Disorders, 3rd edition (beta version). Cephalalgia. **33**, 629–808 (2013). 10.1177/0333102413485658 23771276

[3] Vetvik, K. G., Michael B. R. Are menstrual and non-menstrual migraine attacks different? Curr. Pain Headache Rep. **15**, 339 (2011). 10.1007/s11916-011-0212-4 PMC3165119 21584641

[4] Silberstein S, Patel S (2014). Menstrual migraine: an updated review on hormonal causes, prophylaxis and treatment. Expert Opin. Pharmacother..

[5] Ansari M, Karkhaneh A, Kheirollahi A, Emamgholipour S, Rafiee MH (2017). The effect of melatonin on gene expression of calcitonin gene-related peptide and some proinflammatory mediators in patients with pure menstrual migraine. Acta Neurol. Belg..

[6] Ibrahimi K (2015). Reduced trigeminovascular cyclicity in patients with menstrually related migraine. Neurology..

[7] Jin C (2013). Structural and functional abnormalities in migraine patients without aura. NMR Biomed..

[8] Jixin L, Ling Z, Fangfei L (2015). Disrupted resting-state functional connectivity and its changing trend in migraine suffers. Hum. Brain Mapp.

[9] Ashburner J, F. K. J. Voxel-based morphometry—the methods. Neuroimage **11**, 805–821 (2000). 10.1006/nimg.2000.0582 10860804

[10] Yu-Feng Z (2007). Altered baseline brain activity in children with ADHD revealed by resting-state functional MRI. Brain Develop..

[11] Zang Y, Jiang T, Lu Y, He Y, Tian L (2004). Regional homogeneity approach to fMRI data analysis. Neuroimage..

[12] Shin HE (2008). Headache Impact Test-6 (HIT-6) scores for migraine patients: their relation to disability as measured from a headache diary. J. Clin. Neurol..

[13] Gillian A, H., et al. Measures of adult pain. Arthritis Care Res. **63**, S240–S252 (2011). 10.1002/acr.20543 22588748

[14] Zhaoxia Q, Jingjing S, Xinwei H (2020). Disrupted functional connectivity between sub-regions in the sensorimotor areas and cortex in migraine without aura. J. Headache Pain..

[15] Yaode H, Xiuli Y, Chen Q (2019). Transcatheter patent foramen ovale closure is effective in alleviating migraine in a 5-year follow-up. Front Neurol..

[16] Qiu H, Yingbin Z, Fengzhi W (2020). Impact of right-to-left shunt and transcatheter closure on the clinical features of migraine. Int. J. Neurosci..

[17] Lu H, Gesheng C, Yajuan D (2018). Clinical relevance of atrial septal aneurysm and patent foramen ovale with migraine. World J. Clin. Cases..

[18] Yingqi X, Yuzhu G, Yongsheng G (2016). Effectiveness and safety of transcatheter patent foramen ovale closure for migraine (EASTFORM) trial. Sci Rep..

[19] Lu L, Luopeng Z, Shuiqing Z (2018). Acupuncture as prophylaxis for chronic migraine: a protocol for a single-blinded, double-dummy randomised controlled trial. BMJ Open..

[20] Shunwei L, Yansheng L, Ruozhuo L (2011). Guidelines for diagnosis and treatment of migraine in China. Chin. J. Pain Med..

[21] Ashina M, Bendtsen L, Jensen R, Schifter S, Olesen J (2000). Evidence for increased plasma levels of calcitonin gene-related peptide in migraine outside of attacks. Pain.

[22] Zhi L (2017). Effect of electroacupuncture on urinary leakage among women with stress urinary incontinence: a randomized clinical trial. JAMA.

[23] Andrea M, Karl JF, John A (2005). Voxel-based morphometry of the human brain: methods and applications. Curr. Med. Imaging Rev..

[24] Yan CG, Wang XD, Zuo XN, Zang YF (2016). DPABI: data processing and analysis for (resting-state) brain imaging. Neuroinformatics..

[25] Cui Y, Chen YC (2014). Altered spontaneous brain activity in type 2 diabetes: a resting-state functional MRI study. Diabetes.

[26] Jia Z, Yu S (2017). Grey matter alterations in migraine: a systematic review and meta-analysis. NeuroImage. Clin..

[27] Jia Z, Yu S (2017). Grey matter alterations in migraine: a systematic review and meta-analysis. NeuroImage Clin..

[28] Zeng H (2015). Regional homogeneity (ReHo) changes in new onset versus chronic benign epilepsy of childhood with centrotemporal spikes (BECTS): a resting state fMRI study. Epilepsy Res..

[29] Kaplan JT, Gimbel SI, Harris S (2016). Neural correlates of maintaining one’s political beliefs in the face of counterevidence. Sci. Rep..

[30] Kaplan JT, Gimbel SI, Harris S (2016). Neural correlates of maintaining one's political beliefs in the face of counterevidence. Sci. Rep..

[31] Denckla MB (1999). The human frontal lobes: functions and disorders. Ann. Neurol..

[32] Peyron R (1999). Haemodynamic brain responses to acute pain in humans: sensory and attentional networks. Brain.

[33] Yuan R (2013). Regional homogeneity of resting-state fMRI contributes to both neurovascular and task activation variations. Magn. Reson. Imaging..

[34] Zhu J (2015). Frequency-dependent changes in the regional amplitude and synchronization of resting-state functional MRI in stroke. PLoS ONE.

[35] Jahn A, Nee DE, Alexander WH, Brown JW (2016). Distinct regions within medial prefrontal cortex process pain and cognition. J. Neurosci..

[36] Goadsby PJ, Edvinsson L, Ekman R (1988). Release of vasoactive peptides in the extracerebral circulation of humans and the cat during activation of the trigeminovascular system. Ann Neurol..

[37] Rodríguez OX (2012). Endothelial progenitor cells: a new key for endothelial dysfunction in migraine. Neurology..

[38] Pakalnis A (2016). Migraine and Hormones. Semin. Pediatr. Neurol..

